# Value of the preoperative Model for End-stage Liver Disease score in predicting postoperative outcome in living donor liver transplantation recipients

**DOI:** 10.1186/cc12343

**Published:** 2013-03-19

**Authors:** Y Nassar, K Omar, A Battah, A Mwafy

**Affiliations:** 1 Cairo University, Giza, Egypt

## Introduction

Partial liver grafting predisposes recipients to a unique set of potential technical and anatomic complications that are not prevalent in whole deceased donor grafts. We aimed to assess the preoperative Model for End-stage Liver Disease (MELD) score, postoperative 30-day clinical and laboratory follow-up, and detection of the value of MELD score in predicting 30-day postoperative outcome of LDLT recipients.

## Methods

A prospective multicenter registry involving 142 patients who underwent LDLT from October 2004 to December 2010. Preoperative MELD score was calculated: (0.378×log (bilirubin) (mg/dl) + 1.120×log (INR) + 0.957×log (creatinine) (mg/dl) + 0.643)×10 then rounded to the nearest integer. Postoperative daily recording of all the clinical and laboratory data was done for a period of 30 days.

## Results

The survival rate recorded in our study was 86.62%. The most frequent complications were renal complications (86.6%), pulmonary complications (73.9%), neurological complications (14%), cardiovascular complications (12.6%), infectious complications (13.3%), intra-abdominal infections (10.5%) and immunosuppressant toxicity (7.7%). MELD score proved to be a good predictor of perioperative outcome, patients with MELD score (>12) was associated with less favorable outcome, accuracy was (80.3%).

## Conclusion

MELD score >12 was associated with less favorable outcome with an accuracy of 80.3%.

